# The Potential of Exosomal RNAs in Atherosclerosis Diagnosis and Therapy

**DOI:** 10.3389/fneur.2020.572226

**Published:** 2021-02-11

**Authors:** Wenzhi Yang, Xudong Pan, Aijun Ma

**Affiliations:** The Affiliated Hospital of Qingdao University, Qingdao, China

**Keywords:** atherosclerosis, exosome, RNA, macrophage, endothelial cell

## Abstract

Atherosclerosis is an inflammatory disease that can lead to cardiovascular disorders and stroke. In the atherosclerosis microenvironment, exosomes secreted from various cells, especially macrophage-derived exosomes, play an important role in cell–cell communication and cellular biological functions. In this article, we review previous studies on exosomal RNAs and discuss their potential value in atherosclerosis diagnosis and therapy. Based on our research, we concluded that macrophage exosomes have potential value in atherosclerosis diagnosis and therapy. However, there is a need for future studies to further investigate methods of exosome isolation and targeting.

## Introduction

Atherosclerosis is a chronic inflammatory process that occurs in the arterial wall characterized by lipid accumulation and plaque formation (1). The pathogenesis of atherosclerosis can be divided into three stages: initiation, lesion formation, and thrombosis formation. The initial step is mediated by endothelial cells (ECs) (2). When ECs are subjected to various stimuli, such as changes in blood flow, a focal area of the endothelium will be activated, express adhesion molecules, and recruit leukocytes. ECs in high-lipid environments recruit and bind to monocytes instead of neutrophils; monocytes cross the endothelium after adhering to ECs (3). In response to increased endothelial permeability and changes in the extracellular matrix composition, low-density lipoproteins accumulate in the focal endothelium. After engulfing lipids, monocytes are transformed into foam cells and accumulate in the lesion area. Lesion progression is also associated with extracellular matrix deposition, migration, and proliferation of smooth muscle cells, and formation of a necrotic core (4). If the atherosclerotic plaque is large enough to restrict blood flow, clinical manifestations will occur. Plaque rupture can also lead to myocardial or ischemic stroke, which is associated with collagen-poor fibrous caps (5).

Exosomes are small extracellular vesicles (EVs) with diameters ranging from 40 to 200 nm. Recently, exosomes have become increasingly popular in the scientific community for their great potential for drug delivery. Exosomes are secreted by many cell types and exist in most bodily fluids (6). They play a vital role in cell–cell communication. The contents of exosomes include proteins, lipids, nucleic acids, and small non-coding RNAs such as microRNAs (miRNAs). Among the exosome contents, miRNAs have gained increased attention recently for their potential role in disease diagnosis and treatment. Previous studies suggest that miRNAs have a therapeutic effect in various diseases. The major challenge of using synthetic miRNAs for disease therapy is that they are easily degraded by ribonucleases in plasma. However, this instability can be overcome by inserting them into exosomes. Therefore, exosomes are attractive as a drug-delivery vehicle (7).

Exosomes can serve as carriers across different biological barriers, thereby transferring drugs or biological molecules to target cells. A study suggested that brain EC-derived exosomes can pass the blood-brain barrier (8). Circulating miRNAs have been used as diagnostic biomarkers in atherosclerosis (9). However, different cells might secrete the same miRNAs, which makes it quite difficult to identify their source. However, exosomes carry markers from their source cells. Thus, using these markers to identify the source cells of exosomes and analyzing changes in the content of exosomes can provide information about their parent cells, highlighting the events that occurred in the diseased or disease-causing cells (10). Nevertheless, several issues remain. In particular, exosome isolation is time-consuming and difficult to reproduce. This makes it difficult to compare results between laboratories, improve our knowledge of exosomal physiological functions, and correlate their features with pathological outcomes.

Considering the potential for exosomes to facilitate disease diagnosis and treatment, herein we review recent studies of miRNAs in atherosclerosis (AS)-associated cell-derived exosomes (such as those derived from ECs and macrophages) and in exosome-based therapy or diagnosis of atherosclerosis. This review summarizes our current understanding of the role of exosomal RNAs in atherosclerosis.

## EV Families

Wolf first identified EVs in 1967. Initially, EVs were recognized as platelet-derived particles in plasma, so Wolf chose the name “platelet dust” (18, 19). In 1980, EVs were isolated from rectal adenoma microvillus cells (20) and were reported as “virus-like particles” in cell culture (21). These vesicles were also referred to as “prostasomes” (22). An ultrastructure study in 1983 suggested that these vesicles were released by the fusion of multi-vesicular bodies (MVBs) with the cell membrane in reticulocytes during the differentiation process (23, 24). In 1987, Johnstone's group (25) reported that these vesicles originated from reticulocytes and that “the externalization of these vesicles may be a mechanism for shedding of specific membrane functions, which are known to diminish during maturation of reticulocytes to erythrocytes.” In Johnstone's paper, these vesicles were termed “exosomes.”

According to their source cells and tissues, EVs can be called prostasomes (from the prostate), dexosomes (from dendritic cells), oncosomes (from tumor cells), synaptic vesicles (from nerve cells), or cardiosomes (from cardiac muscle cells). Based on their possible functions, they can be named matrix vesicles, argosomes, or tolerosomes (26, 27). According to their size, biological character, and biogenesis mechanism, there are three main types of EVs: apoptotic bodies, microvesicles (also known as ectosomes), and exosomes. Different kinds of EVs have different biogenesis and releasing mechanisms, markers, and contents.

Apoptotic bodies, ranging from 500 to 2,000 nm, are released when apoptosis occurs. Markers of apoptotic bodies contain phosphatidylserine and genomic DNA. Microvesicles are in the range of 200–1,000 nm, while exosomes are 40–200 nm. Microvesicles and exosomes serve as transporters of mRNA, miRNAs, and proteins to target cells. Microvesicle markers include integrins, selectins, and CD40, and markers of exosomes are tetraspanins, apoptosis-linked gene 2-interacting protein X, and tumor susceptibility gene 101 (28, 29). In general, the naming of EVs is very complex because there are no common markers to identify different kinds of EVs. Thus, the nomenclature of different EVs remains controversial (30). The International Society for Extracellular Vesicles advises the use of physicochemical properties, such as size, density, or biochemical components, to identify different EVs (31).

## Exosome Biogenesis

Exosome biogenesis is mediated by the endosome system. There are three stages in exosome biogenesis. First, the plasma membrane invaginates, forming endocytic vesicles, and a number of endocytic vesicles fuse and produce early endosomes. Then, the early endosome membrane invaginates and forms many intraluminal vesicles (ILVs); in this stage, endosomes are also called MVBs. Finally, MVBs merge with the cell membrane, releasing ILVs into the extracellular space; these ILVs are called exosomes (32).

**Figure 1:**
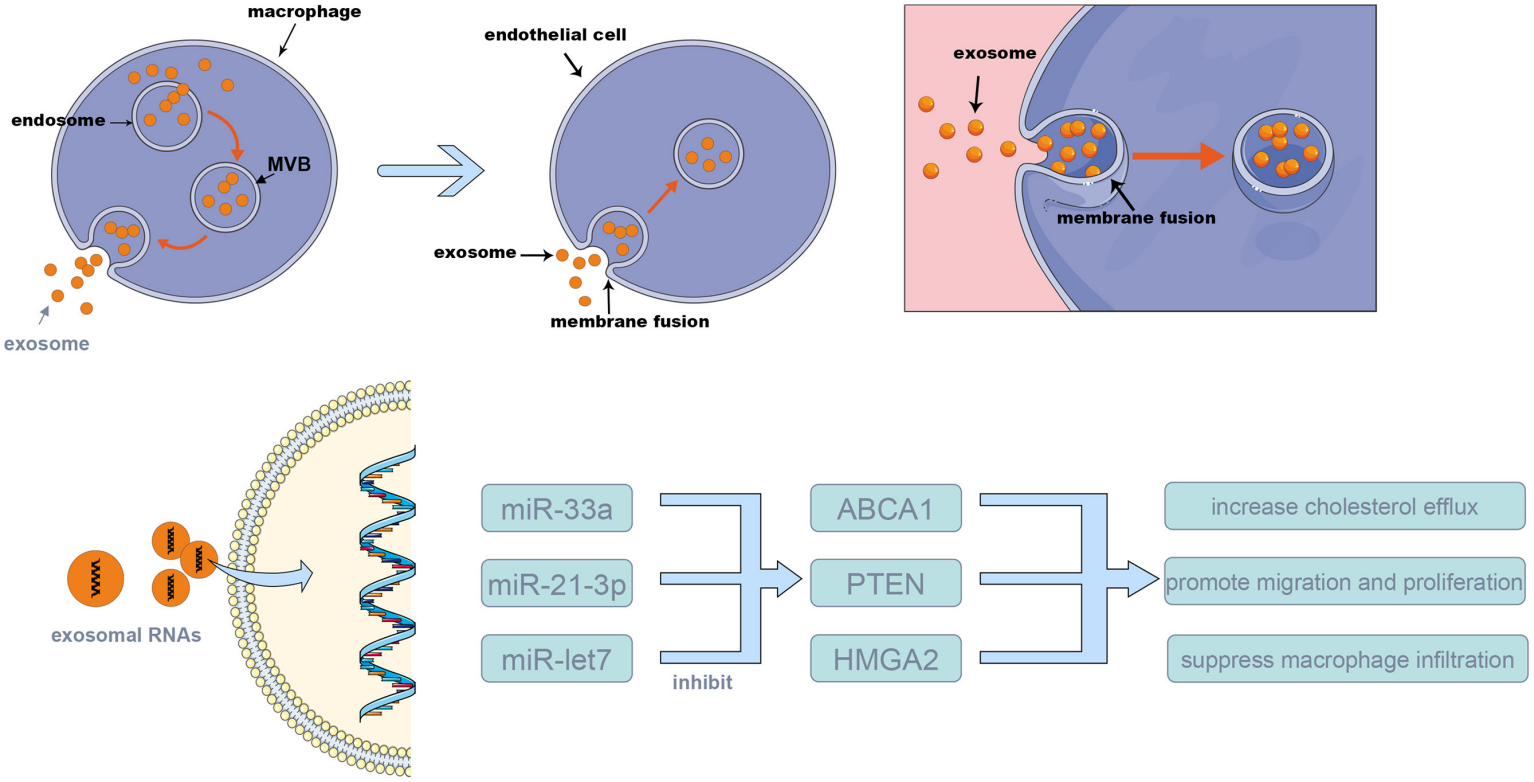
The mechanism of exosome biogenesis and releasing. The multi-vesicular bodies (MVBs) membrane invaginates and forms many exosomes. Then, MVBs merge with the cell membrane, releasing the exosomes into the extracellular space; these exosomes can merge with the membrane of target cells, and release their content to the target cells. The exosomes were released from source cells by MVBs and absorbed by target cells, *via* this procedure, and the exosomal RNAs such as miR-155, miR-33a, and miR-505 were transferred into target cells and take the biological effects (shown in Table 1).

Various proteins are involved in the biogenesis process, such as the endosomal sorting complex required for transport, CD63, CD81, apoptosis-linked gene 2-interacting protein X, and tumor susceptibility gene 101. Exosome transport in cells relies on the cell skeleton system, and the SNARE protein complex and synaptotagmin family are involved in the exosome release process. The exosome membrane is rich in cholesterol, sphingomyelin, and ceramide. Ceramide plays an important role in the formation of ILVs. Exosomes originate from the cell membrane, and the exosome membrane also contains lipid bilayer structures. However, in the exosome membrane, lipids are located in the membrane randomly, while in the cell membrane, they are asymmetric (33–35) (Figure 1).

## Exosomal RNAs and ECs in Atherosclerosis

Injury to ECs is an initial factor of atherosclerosis. EC dysfunction, such as tight-junction disruption and release of inflammatory cytokines, leads to macrophage accumulation and inflammation at the site of injury. Various stimulations, such as changes in blood flow, can lead to EC dysfunction (2, 36). There is no clear conclusion that exosome-mediated cell–cell communication plays a good or bad role in the atherosclerosis process. Zheng et al. found that Krüppel-like factor 5-overexpressing vascular smooth muscle cells (VSMCs), which express a high level of miRNA-155, can secrete miRNA-155-containing exosomes, leading to the impairment of endothelial integrity and facilitating atherosclerosis plaque formation (11). VSMC-derived exosomes can be absorbed by ECs and can regulate EC function, suggesting that exosomes play an important role in communication between VSMCs and ECs. Gao et al. demonstrated that mature dendritic cells contribute to endothelial inflammation and increased atherosclerosis and that exosomes are involved in this process (37). They found that exosomes isolated from bone marrow dendritic cell culture medium increase the inflammation of ECs *via* the NF-κB pathway. They also found that exosomes injected intravenously can be absorbed by ECs *in vivo* and that tumor necrosis factor-α on the exosome membrane triggers NF-κB in ECs.

**Table 1:** The associated RNAs in atherosclerosis.

**References**	**Exosomal RNA**	**Target Molecule**	**Effect**
Zheng et al. (11)	miR-155		Impairment of endothelial integrity
Stamatikos et al. (12)	miR-33a	ABCA1	Increase cholesterol efflux
Chen et al. (13)	miR-505		Induce neutrophil extracellular trap
Gao et al. (14)	lncRNA MALAT1		Promote neutrophil extracellular trap
Zhu et al. (15)	miR-21-3p	PTEN	Promote VSMC migration and proliferation
Li et al. (16)	miR-let7	HMGA2	Suppress macrophage infiltration
Nguyen et al. (17)	miR-146a		Promote macrophage adhesion

Cholesterol accumulation is also a significant cause of atherosclerosis. Stamatikos et al. suggested that EC-derived exosomal miRNA-33a increases macrophage and smooth muscle cell cholesterol efflux by silencing ABCA1. The researchers decreased miRNA-33a expression levels in EC-derived exosomes by constructing a helper-dependent adenovirus to transfect ECs and used these exosomes to treat macrophages and smooth muscle cells (12). Another *in-vitro* study confirmed that ABCA1 overexpression in ECs enhances ApoAI-mediated cholesterol efflux, the results showed that the ABCA1 protein in macrophages and smooth muscle cells was increased by 1.6–2.2-fold and that cholesterol efflux was increased by 1.4–1.6-fold (38). Besides, Stamatikos also reported that transduction of carotid artery ECs with a helper-dependent adenoviral (HDAd) vector expressing apo A-I reduced the atherosclerosis lesion growth (39). These researches demonstrated that modification of the exosome content to regulate the genes expression of target cells may be a new strategy for treating atherosclerosis.

Li et al. found that EC-derived exosomes can be internalized by VSMCs and can inhibit neointima formation and the phenotypic switch of VSMCs. However, activation of EC CD137 signaling attenuates the repressive effects of EC-derived exosomes (40). There are also studies suggesting that the content of EC-derived exosomes changes after treatment with oxidized low-density-lipoprotein (LDLs). For example, Chen found that exosome-encapsulated miR-505 from oxidized LDL-treated ECs induces neutrophil extracellular trap (NET) formation, which aggravates atherosclerosis (13). Mature dendritic cell-derived exosomes participate in the process of endothelial inflammation and atherosclerosis through the membrane tumor necrosis α-mediated NF-κB signaling pathway. Zhong et al. demonstrated that exosomes derived from mature dendritic cells increase adhesion molecule expression by activating the NF-κB pathway in ECs; however, the researchers also showed that ECs are resistant to a second stimulation by these exosomes. This suggests that a negative feedback loop of inflammation regulation occurs *via* dendritic cell-derived exosomes (41).

Exosomal long non-coding RNAs (lncRNAs) also play a role in atherosclerosis. Gao et al. found that human umbilical vein EC exosomal MALAT1 expression increases when the cells are treated with oxidized LDLs. Furthermore, oxidized LDL-treated human umbilical vein EC-derived exosomes were shown to aggravate atherosclerosis by promoting neutrophil NET formation, which is mediated by exosomal MALAT1 (14).

## Exosomal RNAs and Macrophages in Atherosclerosis

When ECs are activated and express adhesion molecules, they recruit leukocytes, mainly monocytes. After the monocytes pass across the vascular wall, they transfer into macrophages, releasing cytokines that mediate focal inflammation. The communication between macrophages, ECs, and VSMCs is complicated, and evidence shows that exosomes are involved in this process. To investigate the treatment effect of exosomes in atherosclerosis, Pu et al. isolated exosomes from M2 macrophages and then electroporated the exosomes with hexyl 5-aminolevulinate hydrochloride. After systemic administration, these exosomes exhibit an anti-inflammatory effect *via* their surface-bonded chemokine receptors and the release of anti-inflammatory cytokines secreted from anti-inflammatory M2 macrophages. Furthermore, exosomal hexyl 5-aminolevulinate hydrochloride induces carbon monoxide and bilirubin, which further strengthens the anti-inflammatory effects and aggravates atherosclerosis (42).

According to Zhu et al., when macrophages are stimulated by nicotine, miRNA-21-3p expression in macrophage-derived exosomes increases, and these exosomes accelerate atherosclerosis by mediating VSMC migration and proliferation *via* the miRNA-21-3p/PTEN pathway (15). Zhang's study showed that miRNA-146 in macrophage-derived exosomes increase when macrophages are treated with oxidized LDLs, which promotes reactive oxygen species and NET release by targeting superoxide dismutase 2, finally aggravating atherosclerosis (43). Li et al. found that treatment with mesenchymal stem cell-derived exosomes decreases the atherosclerotic plaque size in ApoE^−/−^ mice, inhibits the expression in macrophages of increased M2 markers and lipopolysaccharide-induced M1 markers, promotes M2 macrophage polarization through the miR-let7/HMGA2/NF-κB pathway, and suppresses macrophage infiltration *via* the miR-let7/IGF2BP1/PTEN pathway in plaques, which attenuates the progression of atherosclerosis (16). Oxidized-LDL-stimulated macrophages can attenuate the growth and tube formation of ECs (44). MiRNA-146a from atherogenic macrophage-derived exosomes can inhibit cell migration and promote macrophage adhesion in the vascular wall (17) and can accelerate the process of atherosclerosis.

Research has suggested that obesity is associated with atherosclerosis. Xie found that adipose tissue-derived exosomes from mice fed a high-fat diet regulates macrophage foam cell formation and polarization, suggesting that there is an association between adipose tissue and atherosclerosis in obesity (45). Chen et al. found that lncRNA growth arrest-specific 5 (GAS5) is increased in plaques of atherosclerosis patients and animals. After oxidized LDL stimulation, lncRNA GAS5 is also increased in THP-1 cell-derived exosomes. EC apoptosis was promoted by LncRNA GAS5-overexpressing THP-1 cell-derived exosomes but inhibited by lncRNA GAS5-knockdown THP-1 cell-derived exosomes (46). This suggests that atherosclerosis can be alleviated by regulating the apoptosis of macrophages and ECs *via* exosomes.

## Potential Value of Macrophage Exosomal miRNAs in Atherosclerosis

Macrophages play an important role in the mechanism of atherosclerosis. Macrophages are attracted to the injured endothelium and are the main cells that secrete various inflammatory factors to regulate EC functions. The inflammatory environment could alter microRNA profile in exosomes secreted from macrophages, which could give us a better understanding of the mechanism of disease, the altered microRNAs could be a biomarker for diagnosis (47). These exosomes serve as the carrier of miRNAs among macrophages and between macrophages and ECs. The studies previously discussed suggest that macrophage-derived exosomes regulate EC functions *via* absorption by ECs. Importantly, various miRNAs can regulate the atherosclerosis process, such as miR-21. MiR-21 is the most abundant miRNA in macrophages and can regulate macrophage expression of inflammatory cytokines, such as IL-10 (48–50). Thus, modifying miR-21 in macrophage-derived exosomes has the potential to exert a therapeutic effect in patients with atherosclerosis. In addition, macrophage-derived exosomes can regulate VSMC functions, such as proliferation, providing another strategy for treating atherosclerosis. Compared with serum RNAs, exosomal miRNAs and lncRNAs are more stable, and changing their contents in exosomes may be more valuable for treating atherosclerosis.

## Conclusion

In summary, we believe that macrophage exosomes have potential value in atherosclerosis diagnosis and therapy. However, there are still some issues that need to be resolved, and future studies should further investigate methods of exosome isolation and targeting.

## Author Contributions

WY drafted the manuscript. AM and XP contributed to revise the manuscript. All authors contributed to the article and approved the submitted version.

## Conflict of Interest

The authors declare that the research was conducted in the absence of any commercial or financial relationships that could be construed as a potential conflict of interest.
